# The synergistic activity of SBC3 in combination with Ebselen against *Escherichia coli* infection

**DOI:** 10.3389/fphar.2022.1080281

**Published:** 2022-12-15

**Authors:** Hao Chen, Qianqian Lu, Haoyue An, Juntong Li, Shuchu Shen, Xi Zheng, Wei Chen, Lu Wang, Jihong Li, Youqin Du, Yueqing Wang, Xiaowen Liu, Marcus Baumann, Matthias Tacke, Lili Zou, Jun Wang

**Affiliations:** ^1^ The Second People’s Hospital of China Three Gorges University, Yichang, Hubei, China; ^2^ The Second People’s Hospital of Yichang, Yichang, Hubei, China; ^3^ Hubei Key Laboratory of Tumor Microenvironment and Immunotherapy, College of Basic Medical Sciences, China Three Gorges University, Yichang, Hubei, China; ^4^ The Institute of Infection and Inflammation, College of Basic Medical Sciences, China Three Gorges University, Yichang, Hubei, China; ^5^ The School of Chemistry, University College Dublin, Belfield, Dublin, Ireland; ^6^ The People’s Hospital of China Three Gorges University, Yichang, Hubei, China

**Keywords:** SBC3, ebselen (PubChem CID: 3194), *Escherichia coli*, thiol-dependent redox system, peritonitis, redox homeostasis

## Abstract

*Escherichia coli* ranks as the number one clinical isolate in the past years in China according to The China Antimicrobial Surveillance Network (CHINET), and its multidrug-resistant (MDR) pathogenic strains account for over 160 million cases of dysentery and one million deaths per year. Here, our work demonstrates that *E. coli* is highly sensitive to the synergistic combination of SBC3 [1,3-Dibenzyl-4,5-diphenyl-imidazol-2-ylidene silver (I) acetate] and Ebselen, which shows no synergistic toxicity on mammalian cells. The proposed mechanism for the synergistic antibacterial effect of SBC3 in combination with Ebselen is based on directly inhibiting *E. coli* thioredoxin reductase and rapidly depleting glutathione, resulting in the increase of reactive oxygen species that cause bacterial cell death. Furthermore, the bactericidal efficacy of SBC3 in combination with Ebselen has been confirmed in mild and acute peritonitis mice. In addition, the five most difficult to treat Gram-negative bacteria (including *E. coli*, Acinetobacter baumannii, Enterobacter cloacae, *Klebsiella pneumoniae*, and *Pseudomonas aeruginosa*) are also highly sensitive to a synergistic combination of SBC3 and Ebselen. Thus, SBC3 in combination with Ebselen has potential as a treatment for clinically important Gram-negative bacterial infections.

## Introduction

The discovery of antibiotics is considered as the most important achievement in the history of pharmacology (Moser et al., 2019). During the past 8 decades since the introduction of penicillin, several classes became available including β-lactam antibiotics, aminoglycosides, tetracyclines, macrolides, lincomycin, vancomycin, bacitracin, and others that act through mechanisms affecting the bacterial cell wall, the cell membrane, nucleic acid, and protein synthesis (Zinner, 2007; Zou et al., 2017). Appropriate use of antibiotics could save over 160 million cases of dysentery and one million deaths from pathogenic strains every year around the world (Wirth et al., 2006). However, through a variety of mechanisms, including hotspot mutations that are driven by positive selection (Chattopadhyay et al., 2009; Geurtsen et al., 2022) many bacteria are developing antibiotic resistance (Machowska and Stalsby Lundborg, 2018). At present, infections still kill an unacceptable number of patients, even in developed countries with easy access to most antibiotics (Lindstedt et al., 2018; Santos et al., 2020; Geurtsen et al., 2022). Thus, new approaches towards antibacterial chemotherapeutics are highly needed if we are to survive the increasing rates of antibiotic resistance predicted for the near future.

Recognized as a novel antibiotic strategy, inhibition of the bacterial thiol-dependent redox system (TDRS) is gaining momentum (Ren et al., 2020). TDRS includes the thioredoxin (Trx) and glutathione (GSH)/glutaredoxin (Grx) systems. The Trx system consists of Trx and thioredoxin reductase (TrxR); while the GSH/Grx system includes GSH, glutathione reductase (GR) and Grx. As the major antioxidant systems, Trx and GSH/Grx have been found to be able to transfer the electrons crossly to provide electrons to a large range of enzymes including ribonucleotide reductase (RNR), i.e. TrxR could maintain the level of reduced Trx, Grx and RNR; meanwhile, GSH/Grx could do the same and GSH, Trx and RNR (Lu and Holmgren, 2014). Thus, two systems serve as a backup system for each other to maintain the redox homeostasis, participating in cellular signal transduction and regulation, DNA and protein synthesis and repair, affecting bacterial survival and death, activation, and proliferation (Lu and Holmgren, 2014; Ren et al., 2018; Ren et al., 2020).

As promising bactericidal leads N-heterocyclic carbene (NHC)-silver(I) complexes, such as SBC3 [1,3-dibenzyl-4,5-diphenyl-imidazol-2-ylidene silver(I) acetate], possess a reversible, high affinity for inhibition of bacterial thioredoxin reductase (bTrxR); while they have no significant inhibitory effect on mammalian (mTrxR) (O'Loughlin et al., 2021), making it a potential antibacterial candidate. However, although our previous assays showed that SBC3 can efficiently inhibit bacterial cell growth (O'Loughlin et al., 2021; O'Beirne et al., 2021; Sharkey et al., 2012), the high mammalian toxicity of SBC3 with unknown mechanisms restricted its application.

Ebselen (EbSe), is a seleno-organic drug targeting bTrxR as shown in our previous work (Lu et al., 2013; Dong et al., 2019). Clinical trials show that Ebselen is also a promising compound in treating numerous human diseases, including hearing loss (Kil et al., 2017), mania and hypomania (Sharpley et al., 2020) and impulsivity and emotional processing (Masaki et al., 2016), indicating that it possesses both high human tolerance and low toxicity.

*E. coli* is one of the most ubiquitous and pathogenically versatile bacterial organisms (Abbas et al., 2004; Geurtsen et al., 2022). It causes various infections, such as diarrhea, hemorrhagic cystitis, pyelonephritis, urinary tract infections, although not all strains are pathogenic to humans (Santos et al., 2020; Kobayashi et al., 2021). The China Antimicrobial Surveillance Network (CHINET) showed that *E. coli* ranks as the number one clinical isolate in 2021 (18.96%), and many isolates are highly resistant to ampicillin (84.2%), piperacillin (72.1%), ciprofloxacin (57.1%), cefuroxime (55.6%), levofloxacin (53.6%), ceftriaxone (53.8%) and trimethoprim-sulfamethoxazole (TMP-SMZ, 53.5%). Therefore, multidrug-resistant (MDR) *E. coli* still lacks the effective means for control and therefore novel antibiotics are urgently needed.

Here, we present the combination of the silver-containing antibiotic SBC3 with Ebselen exhibiting significant and selective synergistic toxicity on bacteria over mammalian cells by allowing to use a decreased amount of the antibacterial candidate SBC3. The antibiotic strategy is based on disrupting TDRS and up-regulation of ROS. This selective toxicity should facilitate the potential systemic medical application of SBC3 with Ebselen in the prophylaxis and treatment of MDR *E. coli* through the addition of further research and clinical trials involved.

## Material and methods

### Bacterial strains

*Escherichia coli* DHB4, *Acinetobacter baumannii* ATCC 19606, *Enterobacter cloacae* ATCC 700323*, Klebsiella pneumoniae* ATCC 700603, and *Pseudomonas aeruginosa* ATCC 27853 strains were purchased from China General Microbiological Culture Collection Center (CGMCC) and were cultured in Luria Bertani (LB) and stored in the logarithmic phase with 20% glycerin at −80°C in our lab.

*E. coli* multidrug-resistant (MDR) strain BC1 was isolated from patients with UTI at First Clinical Hospital of Yichang (the First College of Clinical Medical Science, China Three Gorges University), with an approval and written informed consent for research from the Ethics Committee of the First Affiliated Hospital of China Three Gorges University and informed consent of the patient (Wang et al., 2020).

### Reagents

1,3-Dibenzyl-4,5-diphenyl-imidazol-2-ylidene silver (I) acetate was obtained from the School of Chemistry, University College Dublin, Ireland. 2-phenyl-1,2-benzisoselenazol-3(2H)-one was purchased from Selleck Chemicals. MEF cell line (embryonic fibroblast cells), and S-KN-SH (human neuroblastoma strain) were purchased from China National Collection of Authenticated Cell Cultures. Protease inhibitor cocktail and 2′,7′-Dichlorodihydrofluorescein diacetate (H_2_DCF-DA) were purchased from MedChemExpress. Iodacetamide (IAM) and Penicillin-streptomycin solution were purchased form ThermoFisher. CellTiter-Glo^®^ for ATP assay was purchased from Promega. Bacterial RNA Extraction Kit, 2× Universal Blue SYBR Green qPCR Master Mix, and SweScript All-in-one First-Strand cDNA Synthesis SuperMix for qPCR were purchased from Vazyme. LB medium was purchased from EMD Millipore. *E. coli* DHB4 Trx protein and anti-*E. coli* Trx1 polyclonal antiserum were purchased from Santa Cruz, IgG2a mouse monoclonal antibody was obtained from VIROGEN, rabbit anti-sheep IgG-HRP and Anti-Dnak antibodies were from Santa Cruz, protease inhibitor cocktails were from Roche, and all the other reagents were from Sigma-Aldrich.

### Toxicity analysis of Ebselen and SBC3 in combination against mammalian cells

MEFs and S-KN-SH were cultured in DMEM medium supplemented with 10% (v/v) fetal bovine serum, 50 units/mL penicillin-streptomycin solution were added at 37°C in a 5% CO_2_ incubator. Cells were seeded in 96 micro-well plates and grown till 80% confluency. Cells were further treated with a serial combination of 0–8 μg/ml SBC3 and Ebselen (0.55, 1.09, 2.19, and 2.72 μg/ml Ebselen equal to 2, 4, 8, 10 µM Ebselen, separately) for 48 h, and the cell toxicity was detected by ATP assay as described by manufacture.

### The synergistic antibacterial effect of SBC3 in combination with Ebselen on the growth of five gram-negative bacteria

There are five Gram-negative pathogen species: *E. coli* DHB4, uropathogenic *E. coli* BC1, *A. baumannii* ATCC 19606, *E. cloacae* ATCC 700323*, K. pneumoniae* ATCC 700603, and *P. aeruginosa* ATCC 27853. All cultures were grown until an A_600_ of 0.4 and at 37°C, 180 rpm and further diluted in 1:1,000 times in LB medium and further treated by a serial combination of SBC3 and Ebselen for 24 h, and the A_600_ of bacterial culture was detected to calculate bacterial viability. Bacterial viability was measured using Ultraviolet-visible spectrophotometry (UV-Vis) at A_600_. The mean OD of four wells in the indicated groups was used to calculate the percentage of bacterial viability as follows: percentage of bacterial viability = (A_treatment_ − A_blank_)/(A_control_ − A_blank_) × 100% (where, A = absorbance). Values were plotted by averaging duplicate wells.

The synergism of SBC3 and Ebselen was determined using the Bliss Independence Model, which calculates a degree of synergy using the following formula: S = (f_X0_/f_00_) (f_0Y_/f_00_) − (f_XY_/f_00_), where f_XY_ refers to *E. coli* DHB4 growth rate in the presence of the SBC3 and Ebselen in combination at a concentration X, for SBC3, and Y for Ebselen; f_X0_ and f_0Y_ refer to *E. coli* DHB4 growth rates in the presence of the individual SBC3 and Ebselen at a concentration of X and Y, respectively; f_00_ refers to *E. coli* DHB4 growth rate in the absence of SBC3 and Ebselen; and S corresponds to the degree of synergy.

### SBC3 in combination with Ebselen on bacterial morphology

*E. coli* DHB4 was grown until an A_600_ of 0.4 at 37°C, 180 rpm and treated with 6 μg/ml SBC3 and 21.6 μg/ml Ebselen for 30 min. Cells were obtained by 4°C, 12,000 rpm, 15 min centrifugation and further fixed with 2.5% glutaraldehyde. Bacterial cells were stained by 2% uranyl acetate and dropped to the surface of copper wire mesh for the sample preparation. The morphology of DHB4 cells was observed by transmission electron microscopy (TEM, Hitachi H-7500).

### Measurement of ROS production in *E. coli* DHB4 cells

*E. coli* DHB4 cells were cultured until an A_600_ of 0.4 at 37°C, 180 rpm and incubated with 6 μg/ml (10 μM) SBC3, 21.6 μg/ml (80 μM) Ebselen, and 6 μg/ml SBC3 and 21.6 μg/ml Ebselen for 30 min respectively. The DHB4 cells were stained at 37°C with 10 μmol/L H_2_DCF-DA. After 30 min incubation, the intracellular ROS production level was quantified by flow cytometry (BECKMAN COULTER, CytoFLEX). Briefly, an FSC/SSC plot was draw with both axis are in log mode. Then the samples were run at low-rate settings to acquire at least 5,000 cells per sample. A gate was created on the unstained sample and was applied to the rest to acquire fluorescent data from the stained sample and calculate the statistics.

### Measurement of Trx and TrxR activities and GSH amount in *E. coli* DHB4 cells

*E. coli* DHB4 cells were cultured until an A_600_ of 0.4 at 37°C, 180 rpm and incubated with 6 μg/ml (10 μM) SBC3 and 21.6 μg/ml (80 μM) Ebselen, 6 μg/ml SBC3 and 24.3 μg/ml (90 μM) Ebselen, 6 μg/ml SBC3 and 27.0 μg/ml (100 μM) Ebselen for 30 min respectively. DHB4 cells were obtained by 4°C, 5,000 rpm, 3 min centrifugation, and treated by a protease inhibitor cocktail (in 50 mmol/L Tris-HcL-EDTA buffer, pH 7.4). The DHB4 cells were further disrupted by sonication (30 W, 10 min), and the supernatants were obtained by 4°C, 12,000 rpm, 20 min centrifugation. The total protein has been detected by BCA assay and normalized to 20 μg, and the Trx/TrxR activities and GSH amount assays were performed in a 96-well plate by DTNB assay as described previously (Zou et al., 2017). The activity of the untreated group was 100%.

DTNB experiments were carried out with 96 micro-well plates in the reaction mixture containing pH 7.5 50 mM Tris-HCl, 200 μM NADPH, 1 mM EDTA, 1 mM DTNB, in the presence of 5 μM *E coli* Trx. The UV-vis at A_412_ was measured to represent TrxR activity. The Trx activity was detected by DTNB assay coupled with 100 nM *E coli* TrxR instead of 5 μM Trx in the solution. To measure GSH total amount, 25 μg of the cell lysates was added in the solution containing 50 nM GR, pH 7.5 50 mM Tris-HCl, 200 μM NADPH, 1 mM EDTA, 1 mM DTNB.

### Trx1 and proteins S-glutathionylation in *E. coli* DHB4 cells

*E. coli* DHB4 cells were cultured until an A_600_ of 0.4 at 37°C, 180 rpm and incubated with 6 μg/ml (10 μM) SBC3 and 21.6 μg/ml (80 μM) Ebselen, 6 μg/ml SBC3 and 24.3 μg/ml (90 μM) Ebselen, 6 μg/ml SBC3 and 27.0 μg/ml (100 μM) Ebselen for 30 min respectively and were detected by Western blotting assay. After lysis by sonication as described above, the cell lysates were obtained by centrifugation at 12,000 rpm for 20 min and re-suspended in lysis buffer containing 30 mM IAM (protease inhibitor) only for glutathione-protein complexes. Western blotting assay was performed with anti-*E coli* Trx1 polyclonal antiserum (for Trx1) or IgG2a mouse monoclonal antibody (for glutathione-protein complexes). The normalized Trx1 level with respect to the reference DnaK has been presented.

### *trax/trxb/grxa/rnr* mRNA expression in *E. coli* DHB4 cells

*E. coli* DHB4 cells were cultured until an A_600_ of 0.4 at 37°C, 180 rpm and incubated with 6 μg/ml (10 μM) SBC3 and 21.6 μg/ml (80 μM) Ebselen, 6 μg/ml SBC3 and 24.3 μg/ml (90 μM) Ebselen, 6 μg/ml SBC3 and 27.0 μg/ml (100 μM) Ebselen for 30 min respectively. The total RNA was extracted, and further reverse transcribed to cDNA. Real-time fluorescence PCR (qPCR) analysis was performed with a StepOne Plus system. The qPCR primers for the genes of interest, *trxa/trxb/grxa/rnr* and a housekeeping gene *rrsA* were used as the reference and are listed in Supplementary Table S1, and the cycling protocol was briefly listed as follows. An initial denaturation at 95°C for 30 s, 40 cycles at 95°C for 5 s, 61°C for 30 s. A corresponding melting curve was constructed to ensure that there was no contamination. qPCR assay was performed in triplicate for each sample and a no template control was included to rule out contamination and primer-dimers’ formation. The expression fold changes of *trxa/trxb/grxa/rnr* were calculated based on the comparison with *rrsA*.

### The prophylaxis of peritonitis in mice by SBC3 in combination with Ebselen

All animal experiments were performed in accordance with the relevant guidelines and regulations. Sixty healthy male BALB/c mice were randomly divided into four groups (n = 15). Mice were injected i.p. with DMSO, 2 mg/kg SBC3, 25 mg/kg Ebselen, 2 mg/kg SBC3 and 25 mg/kg Ebselen in combination on day 3 and 1 before infection. On day 0, mice were infected i.p. with 100 μL 2 × 10^8^ CFU/mL *E coli* BC1 to construct an acute peritonitis model. Seven days post infection, the overall survival was calculated.

Forty healthy male BALB/c mice were divided into four groups randomly (n = 10). Mice were injected i.p. with DMSO, 2 mg/kg SBC3, 25 mg/kg Ebselen, 2 mg/kg SBC3 and 25 mg/kg Ebselen in combination on the day 3and 1 before infection. On day 0, mice were infected i.p. with 100 μL 2 × 10^7^ CFU/mL *E coli* BC1 to construct a mild peritonitis model. The bacterial load was calculated by counting the colony formation unity (CFU). Mice serum were collected on the day 2, 4, and 6- post-infection and centrifuged at 3,000 rpm for 10 min. The creatinine (Cre), serum urea (UA), alanine transaminase (ALT), and aspartate aminotransferase (AST) were determined by biochemical analyzer (HITACHI 7180) as described previously (Wang et al., 2020; Dong et al., 2022).

### Statistical analyses

Statistical analyses were performed by GraphPad Prism 9.0 (GraphPad Software). Means of data between two groups were contrasted using Student’s *t* test. Data among groups were contrasted using ONE-WAY ANOVA. Sample rates between two groups were tested with chi-square analysis. Overall survival was analyzed by the log-rank (Mantel-Cox) test. *p* values of <0.05 were significant.

## Results

### SBC3 and Ebselen in combination exhibits selective and synergistic toxicity against bacteria over mammalian cells

**FIGURE 1:**
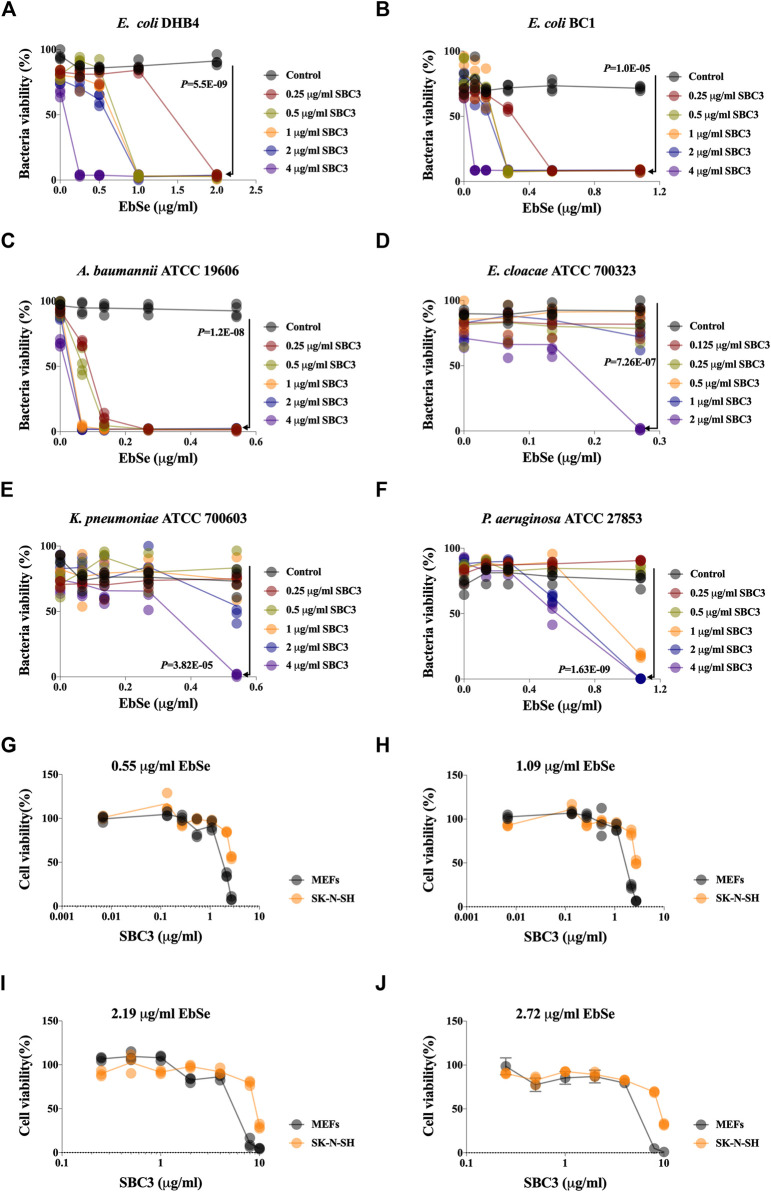
Effects of SBC3 in combination with Ebselen on the growth of bacteria and mammalian cells. **(A–J)** Bacteria overnight cultures were diluted 1:1,000 into 100 μL of LB medium in 96 micro-well plates and treated with 100 μL serial dilutions of SBC3 and Ebselen. **(A)** *E. coli* DHB4. **(B)** *E. coli* BC1. **(C)** *A. baumannii* ATCC 19606. **(D)** *E. cloacae* ATCC 700323. **(E)** *P. aeruginosa* ATCC 27853. **(F)** *K. pneumoniae* ATCC 700603. **(G–J)** The toxicity of SBC3 and Ebselen on the growth of MEFs and S-KN-SH. Cells were treated with serial concentrations of SBC3 and Ebselen for 24 h and the cell toxicity was detected by ATP assay. Data are presented by Student’s *t* test, *p* < 0.05 constitutes statistical significance.

The synergistic effect of SBC3 in combination with Ebselen on the growth of *E. coli* DHB4 was detected by UV-Vis assay at A_600_. The results showed that SBC3 alone inhibited *E. coli* DHB4 growth with a MIC of 25 μg/ml (Supplementary Figure S1A), while the addition of 2.16 μg/ml Ebselen decreased the MIC of SBC3 to 0.25 μg/ml (100 times, Figure 1A) with the *S* degree of 0.77 (Supplementary Table S2). SBC3 alone inhibited *E. coli* BC1 growth with a MIC of 16 μg/ml (Supplementary Figure S1B), while the addition of 0.54 μg/ml Ebselen decreased the MIC of SBC3 to 0.25 μg/ml (64 times, Figure 1B) with the *S* degree of 0.81 (Supplementary Table S2). SBC3 alone inhibited *A. baumannii* ATCC19606 growth with a MIC of 20 μg/ml (Supplementary Figure S1C), while the addition of 0.27 μg/ml Ebselen dramatically decreased the MIC of SBC3 to 0.25 μg/ml (80 times, Figure 1C) with the *S* degree of 0.24 (Supplementary Table S2). SBC3 alone inhibited *E. cloacae* ATCC700323 growth with a MIC of 8 μg/ml (Supplementary Figure S1D), while the addition of 0.27 μg/ml Ebselen decreased the MIC of SBC3 to 2 μg/ml (4 times, Figure 1D) with the *S* degree of 0.77 (Supplementary Table S2). SBC3 alone inhibited *K. pneumoniae* ATCC700603 growth with a MIC of 20 μg/ml (Supplementary Figure S1E), while the addition of 0.54 μg/ml Ebselen decreased the MIC of SBC3 to 4 μg/ml (5 times, Figure 1E) with the *S* degree of 0.62 (Supplementary Table S2). SBC3 alone inhibited *P. aeruginosa* ATCC27853 growth with a MIC of 25 μg/ml (Supplementary Figure S1F), while the addition of 1.08 μg/ml Ebselen decreased the MIC of SBC3 to 2 μg/ml (12.5 times, Figure 1F) with the *S* degree of 1.00 (Supplementary Table S2).

The toxicity of SBC3 and Ebselen on the growth of MEFs and S-KN-SH were also detected. Cells were treated with a serial concentration of SBC3 for 24 h and the cell toxicity was detected by ATP assay. The results showed that SBC3 possesses a dose-dependent toxicity on mammalian cells, the addition of Ebselen will effectively reduce its applicable concentration and will not increase the IC_50_ values of SBC3 against MEFs and S-KN-SH (Figures 1G–J). Thus, the above results indicated that Ebselen and SBC3 in combination showed the synergy of antibacterial effect with the decreased application dose of SBC3.

### SBC3 and Ebselen in combination display strong bactericidal activity against *E. coli*

**FIGURE 2:**
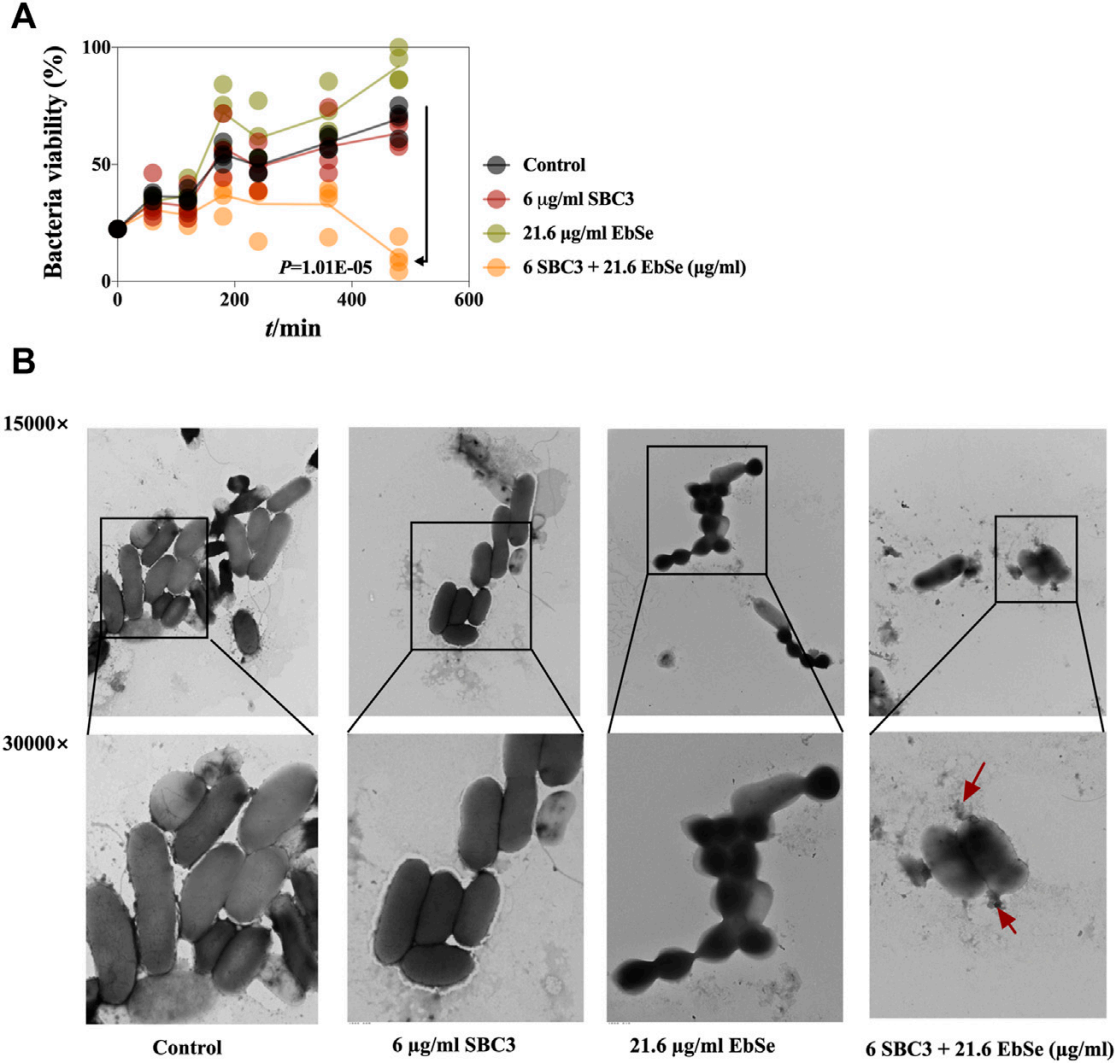
Antibacterial effect of Ebselen and SBC3 on *E. coli* DHB4. *E. coli* DHB4 was cultured until an A_600_ = 0.4 and treated for 30 min with SBC3 and Ebselen. **(A)** Cell viability was represented by measuring A_600_. The growth curves showed a synergistic antibacterial effect of SBC3 and Ebselen on *E. coli* DHB4. Data are presented by Student’s *t* test, *p* < 0.05 constitutes statistical significance. **(B)** Transmission electron microscopy showed the morphology change of DHB4 after the treatment with SBC3 and Ebselen at 300,000× (upper row) or 150,000× (lower row) magnification.

Cell viability was represented by measuring A_600_. The growth curve showed a synergistic antibacterial effect of SBC3 and Ebselen on *E. coli* DHB4. 6 μg/ml SBC3 and 21.6 μg/ml Ebselen inhibited *E. coli* growth 480 min post-treatment (Figure 2A and Supplementary Figure S2). The effect of SBC3 in combination with Ebselen on the morphology of *E. coli* DHB4 was observed by TEM. Normal *E. coli* DHB4 has a smooth surface and an integrated cell membrane and cell wall. By treatment with 6 μg/ml SBC3 and 21.6 μg/ml Ebselen for 30 min, *E. coli* DHB4 cells were deformed, cell membranes and cell walls were ruptured, and overflow of cell contents was detected; meanwhile, although 21.6 μg/ml Ebselen treated *E. coli* DHB4 cells showed morphological changes, there is no obvious rupture of bacterial cells (Figure 2B). All the results showed that the treatment of SBC3 in combination with Ebselen displays strong bactericidal activity against *E. coli* DHB4.

### SBC3 in combination with Ebselen disrupt the intracellular redox homeostasis in *E. coli*

**FIGURE 3:**
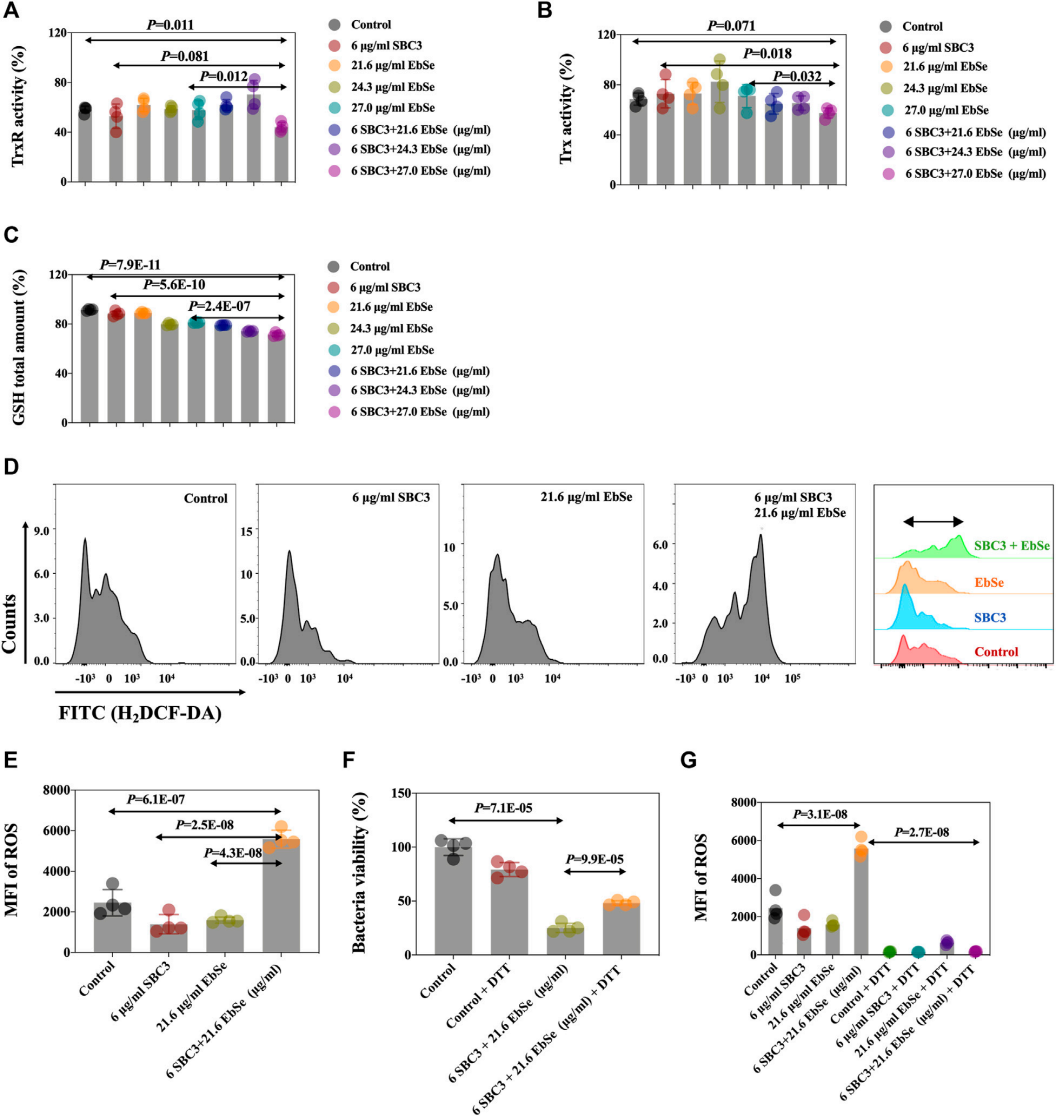
Antibacterial effect of Ebselen and SBC3 on DHB4 targeting TDRS system and producing of ROS. *E. coli* DHB4 was cultured until an A_600_ = 0.4 and treated for 30 min with SBC3 and Ebselen. **(A)** TrxR activity endpoint was detected using DTNB reduction assay in the presence of Trx in DHB4 extracts. **(B)** Trx activity endpoint was detected using DTNB reduction assay in the presence of TrxR in DHB4 extracts. **(C)** GSH activity endpoint was detected using DTNB reduction assay in the presence of GR in DHB4 extracts. **(D,E)** Mean fluorescent intensity (MFI) of H_2_DCF-DA-stained DHB4 was used to detect ROS level by flow cytometry. **(F)** UV-vis at A_600_ has been used to present DTT could save *E. coli* DHB4 from SBC3 and Ebselen treatment. **(G)** Flow cytometry has been used to detect DTT could statistically down-regulate the intracellular ROS production level of *E. coli* DHB4. Data are presented by ONE-WAY ANOVA, *p* < 0.05 constitutes statistical significance.

The effects of SBC3 in combination with Ebselen on bacterial TrxR and Trx activities and the GSH amount in *E. coli* DHB4 were measured by a DTNB assay. All the results showed that the combination of 6 μg/ml SBC3 and 27.0 μg/ml Ebselen could efficiently inhibit the activity of TrxR and deplete the amount of GSH when compared to the control or 27.0 μg/ml Ebselen alone; meanwhile, the activity of Trx1 showed no difference when compared to the control (Figures 3A–C).

The mean fluorescent intensity (MFI) of ROS in *E. coli* DHB4 cells was detected by flow cytometry after being stained by H_2_DCF-DA (Ezraty et al., 2017; Zou et al., 2017). The result showed that the intracellular ROS level in *E. coli* DHB4 cells that were treated with 6 μg/ml SBC3 and 21.6 μg/ml Ebselen was significantly up-regulated when compared with the control (Figures 3D,E). The thiol-containing compound dithiothreitol (DTT) is an organic reducing agent that can remove ROS, and the protective effect of dithiothreitol (DTT) against SBC3 in combination with Ebselen treated *E. coli* DHB4 was evaluated by UV-Vis at A_600_ and FCM, separately. Although 4 mmol/L DTT can effectively rescue *E. coli* DHB4 from the bactericidal effect of 6 μg/ml SBC3 and 21.6 μg/ml Ebselen, DTT at this concentration can be toxic. That is why with the addition of 4 mmol/L DTT, full recovery of *E. coli* DHB4 from SBC3 and Ebselen treatment was not possible (Figure 3F). Meanwhile, the results in Figure 3G showed that 6 μg/ml SBC3 and 21.6 μg/ml Ebselen could cause significantly high MFI of ROS, which also has been statistically reduced with the addition of 4 mmol/L DTT. Both results indicated that the death of *E. coli* DHB4 caused by SBC3 and Ebselen in combination was related to ROS production.

**FIGURE 4:**
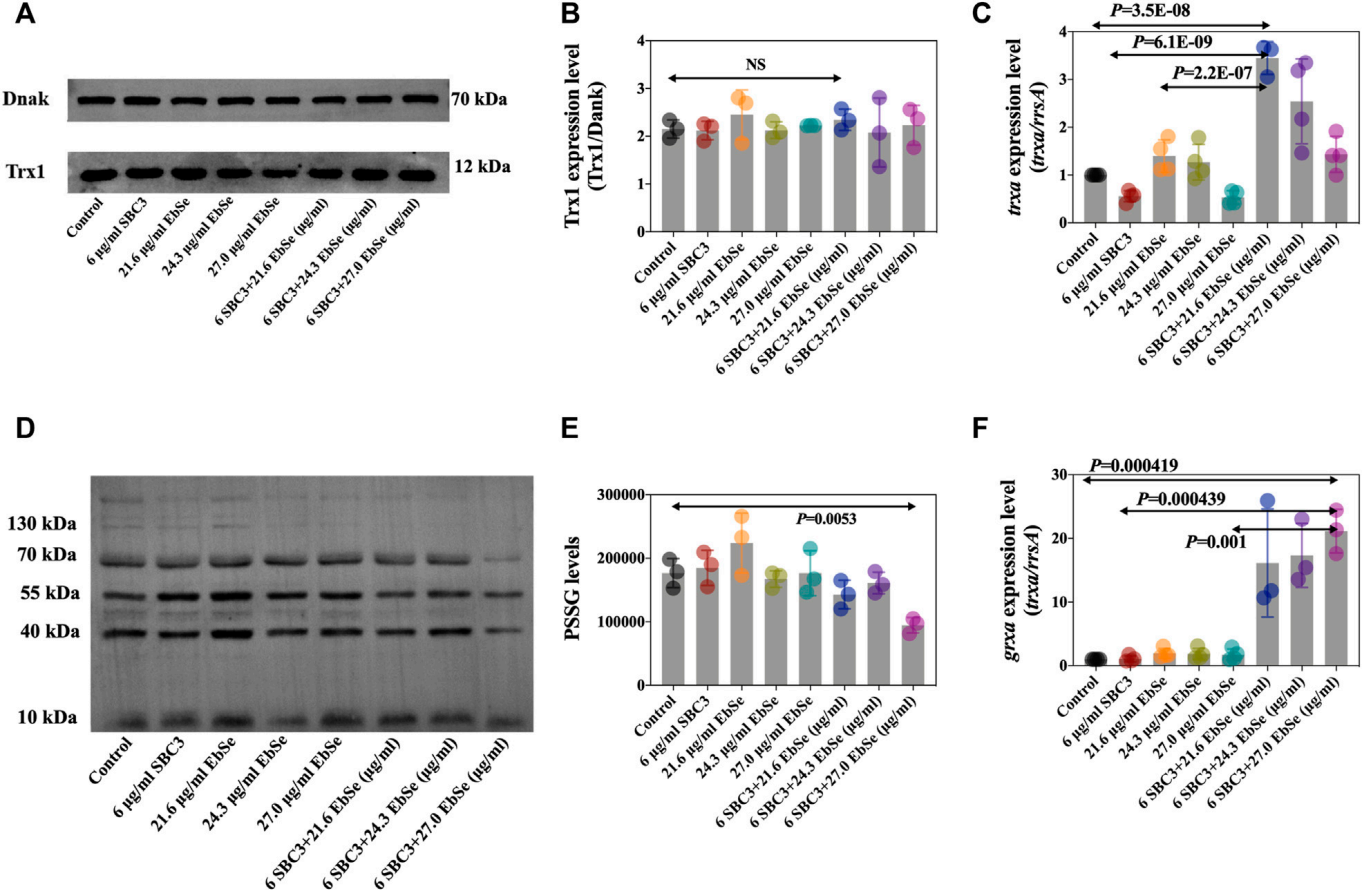
SBC3 with Ebselen in combination directly disrupted bacterial TDRS systems. DHB4 was cultured until an A_600_ = 0.4 and treated for 30 min with serial concentrations of SBC3 and Ebselen. **(A,B)** Trx1 protein level was measured by Western blot. **(C)** *trxa* (Trx1) mRNA expression level was tested by qPCR. **(D,E)** *S*-PSSG protein level was measured by Western blot. **(F)** *grxa* (Grx1) mRNA expression level was tested by qPCR. For Western blot, DnaK was used as a reference; for qPCR, *rrsA* was used as a reference. Data are presented by ONE-WAY ANOVA, *p* < 0.05 constitutes statistical significance.

Whether SBC3 in combination with Ebselen could affect the expression levels of protein Trx1 and S-PSSG was further analyzed by Western blot assay. The results showed that the Trx1 protein expression level was not different by densitometric analysis, indicating the treatment of SBC3 in combination with Ebselen has no influence on Trx1 protein expression (Figures 4A,B). Meanwhile, protein *S*-PSSG was significantly reduced by 6 μg/ml SBC3 in combination with 27.0 μg/ml Ebselen treatment compared to 6 μg/ml SBC3 or 27.0 μg/ml Ebselen alone (Figures 4D,E), which further points to the loss of GSH. Furthermore, the mRNA expression levels of key gens in TDRS were also detected by qPCR. The results show that the relative expression level of *trxa* (encodes Trx1, Figure 4C), *trxb* (encodes TrxR, Supplementary Figure S3A), *grxa* (encodes GR1, Figure 4F) and *rnr* (encodes RNR, Supplementary Figure S3B) in SBC3 in combination with Ebselen treatment group were significantly up-regulated when compared with those for the control. These results show that SBC3 in combination with Ebselen affects the Trx system bioactivity rather than mRNA or protein expressions.

All the results suggest that SBC3 in combination with Ebselen could disrupt *E. coli* intracellular redox homeostasis to perform the bactericidal activity.

### The depletion of *E. coli* BC1 in the peritonitis mice by SBC3 and Ebselen

**FIGURE 5:**
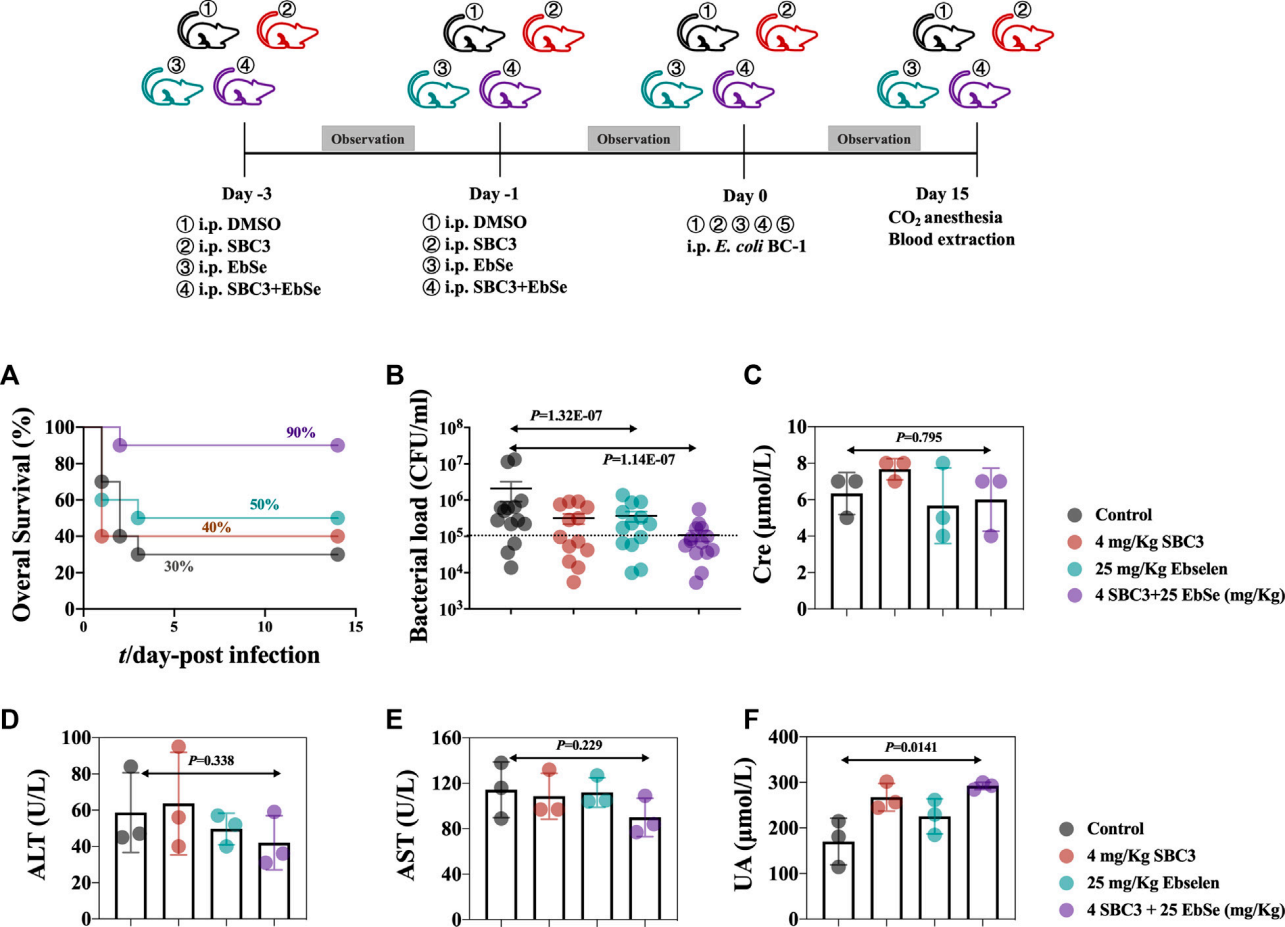
Therapeutic efficacy of SBC3 and Ebselen in treating *E. coli* BC1-induced peritonitis. **(A)** MDR BC1 was cultured overnight, and mice (n = 15) were injected i.p. with DMSO, 2 mg/kg SBC3, 25 mg/kg Ebselen, 2 mg/kg SBC3 and 25 mg/kg Ebselen in combination on the day 1- and 3- before infection. On day 0, mice infected with 100 μL 2 × 10^8^ CFU/ml to construct an acute peritonitis model. Overall survival was observed. Data are presented by log-rank (Mantel-Cox) test, *p* < 0.05 constitutes statistical significance. **(B)** MDR BC1 was cultured overnight, and mice (n = 10) were injected i.p. with DMSO, 2 mg/kg SBC3, 25 mg/kg Ebselen, 2 mg/kg SBC3 and 25 mg/kg Ebselen in combination on the day 1- and 3- before infection. On day 0, mice infected with 100 μL 2 × 10^7^ CFU/ml to construct a mild peritonitis model. The bacterial load was calculated by counting the colonies. Data are presented by chi-square test, *p* < 0.05 constitutes statistical significance. To evaluate the effects of SBC3 in combination with Ebselen on the kidney and liver function of mice, the sera of mice were collected 14 days post-infection, and contents of CRE **(C)**, ALT **(D)**, AST **(E)**, and UA **(F)** were measured. Data are presented by Student’s *t* test, *p* < 0.05 constitutes statistical significance.

Mice were randomly divided into four groups, and i.p. injected with DMSO, 2 mg/kg SBC3, 25 mg/kg Ebselen, 2 mg/kg SBC3 and 25 mg/kg Ebselen in combination on the day 3 and 1 before infection. On day 0, mice were further infected with 100 μL 2 × 10^8^ CFU/ml or 100 μL 2 × 10^7^ CFU/ml to construct acute and mild peritonitis models, respectively. 90% of the mice with acute peritonitis treated with SBC3 in combination with Ebselen survived, compared with 30% in the DMSO group (Figure 5A). Meanwhile, SBC3 and Ebselen in combination led to a significant reduction in bacterial load compared with the DMSO treatment (Figure 5B). Mice treated with SBC3 and Ebselen in combination achieved a 76% reduction, followed by 54% in the SBC3 treatment and 26% in the Ebselen treatment (Figure 5B). All the findings demonstrated the highly effective antibacterial effect of SBC3 and Ebselen in combination against MDR *E. coli* BC1 *in vivo*.

The peripheral blood from different groups of mice were collected, Cre, UA, ALT, and AST in mouse blood serum were detected, and the results showed that SBC3 and Ebselen treatment had no influence on the Cre (Figure 5C), ALT (Figure 5D), and AST levels (Figure 5E). Although there was a slight increase of the UA, the results still fall into the normal level (1.7–8.3 mM, Figure 5F).

Overall, SBC3 and Ebselen treated-mice exhibited the greatest effects in reducing the MDR *E. coli* BC1-caused infection, demonstrating SBC3 and Ebselen may assist the recovery from MDR *E. coli*-caused infection.

## Discussion

The rapid emergence of MDR Gram-negative bacteria poses a great threat to human health and public awareness of an emerging crisis has been highlighted (Mills and Marchaim, 2021). According to the World Health Organization (WHO), *E. coli*, *A. baumannii*, *E. cloacae, K. pneumoniae,* and *P. aeruginosa* are the five most difficult-to-treat Gram-negative members of the so-called ESKAPE, which can be deadly in the clinic, causing life-threatening pneumonia, septicemia, and nosocomial diseases with only last-resort antibiotics to combat them (Hawkey, 2015; Otsuka, 2020).

*N*-heterocyclic silver carbene (NHC-silver) complexes have been recently found to be active agents in treating pathogenic infections, including cystic fibrosis and chronic lung infections (Kascatan-Nebioglu et al., 2006; Hindi et al., 2008). We previously synthesized a new series of p-cyanobenzyl- and benzyl-substituted NHC-silver (Patil et al., 2011), and SBC3 has been identified as the lead compound for the promising inhibition of MRSA in a murine thigh infection model (O'Beirne et al., 2021). However, SBC3 alone has notable mammalian cell toxicity in a dose-dependent manner (Patil et al., 2011). To expand its application as an antibacterial agent, more research has to be performed to reduce its toxicity.

We report here that SBC3 can work synergistically with Ebselen to treat the five most difficult-to-treat Gram-negative bacteria. An initial UV-Vis assay showed the pharmacological synergy of SBC3 and Ebselen, followed by the TEM results that revealed that SBC3 in combination with Ebselen could cause the morphological change (deformation, shrinkage, and content overflow) of *E. coli* DHB4 cells, suggesting the rupture and decomposition of *E. coli* DHB4 cell membrane. The dramatic decrease in MIC of SBC3 against bacteria in the presence of Ebselen makes the potential systemic medical application of silver-containing compounds feasible.

The lethality of current bactericidal antibiotics is accompanied by redox physiology alteration and high amount of ROS generation (Abbas et al., 2004; Kohanski et al., 2010; Van Acker and Coenye, 2017). Known as a highly reactive oxygen-containing molecule, ROS are continuously produced *via* metabolism and eliminated by sophisticated antioxidant systems (Rabinovitch et al., 2017). Excessive amounts of ROS oxidize proteins, lipids, and DNA (Lei et al., 2018), and its intracellular homeostasis plays a vital role in regulating diverse physiological functions in bacterial cells (Zhang et al., 2021). We previously have proven that Ebselen does work synergistically with silver nitrate (Ag^+^) to kill several Gram-negative bacteria including MDR *E. coli* (Zou et al., 2017), *A. baumannii* (Dong et al., 2020), and *Yersinia pseudotuberculosis* (Dong et al., 2022) by disrupting the TDRS and promoting the intracellular redox homeostasis (Zou et al., 2018; Dong et al., 2019). As a silver-based N-heterocyclic carbene, we propose that SBC3 could work with Ebselen in a similar manner.

Here, to determine the enzyme activities and amount in TDRS, DTNB assays were performed. The results showed that the activity of TrxR and the total amount of GSH were both significantly decreased along with the high up-regulation of ROS by SBC3 and Ebselen treatment, indicating that they disrupt redox homeostasis. The most significant finding highlighted here is that Trx1 is not the target of SBC3 and Ebselen in combination when compared with the control group. As the key enzymes in the Trx system, TrxR transfer electrons to Trx to maintain redox homeostasis. It has been noticed that the structure and the electron transfer manner have notable differences between hTrxR and bTrxR (Lu et al., 2013). Meanwhile, as a ubiquitous enzyme, Trx1 is highly conserved among living organisms (Lu and Holmgren, 2014). In our previous studies, we have proven that Ag^+^ or Ag^+^ combined with Ebselen could irreversibly bind to TrxR as well as Trx1 (Zou et al., 2017; Dong et al., 2020; Dong et al., 2022). However, the results here indicate that SBC3 and Ebselen in combination only statistically influence the activity of TrxR. Although the mechanism remains unclear, the results obtained so far still render this drug combination as promising antibacterial agents.

Further, the results showed that the mRNA expression level of *trxa* was increased, and the corresponding protein level of Trx1 was maintained. Although the mRNA expression level showed inconsistency with protein expression level yet combined with the Trx1 activity data, we believe there is a lack of linkage mechanism. The degree to which protein abundance scales with mRNA levels remains an intensely debated topic (Buccitelli and Selbach, 2020). As a well-known phenomenon, total mRNAs poorly correlate to proteins in their abundances as reported (Wang et al., 2013). The relationship between protein and mRNA expression levels informs about the combined outcomes of translation and protein degradation (de Sousa Abreu et al., 2009), transcript levels by themselves are not sufficient to predict protein levels in many scenarios (Liu et al., 2016).

Meanwhile, the S-PSSG expression level significantly decreased after SBC3 and Ebselen treatment, suggesting that the GSH system was severely disrupted by consumption of the GSH pool. We hypothesize that the bacterial cells must pump the oxidized GSH extracellularly since the reaction is too fast for bacteria to reduce it through reductants effectively. However, this hypothesis still needs further studies.

Finally, results from animal experiments indicate that this new antibiotic combination could be considered as a candidate for clinical trials against MDR *E. coli*, and the TDRS targeted by the combination of SBC3 and Ebselen is critical for bacterial survival.

## Conclusion

Our work presented here showcases the synergistic bactericidal effect of SBC3 in combination with Ebselen. This combination can directly inhibit the activity of *E. coli* TrxR, and rapidly deplete GSH, resulting in the up-regulation of the ROS level thus leading to cell death *in vitro*. Further, the protective effect of SBC3 in combination with Ebselen *in vivo* has been confirmed by studying a peritonitis mice model. Thus, SBC3 in combination with Ebselen has the potential to become an emergency antibiotic for the control of clinically important Gram-negative bacteria through the addition of further research and clinical trials.

## Data Availability

The original contributions presented in the study are included in the article/Supplementary Material, further inquiries can be directed to the corresponding authors.
