# Supplementary figures and images for: The synergistic activity of SBC3 in combination with Ebselen against Escherichia coli infection
Source: Front Pharmacol. 2022 Dec 15;13:1080281. doi: 10.3389/fphar.2022.1080281 (PMC9797518; doi:10.3389/fphar.2022.1080281)

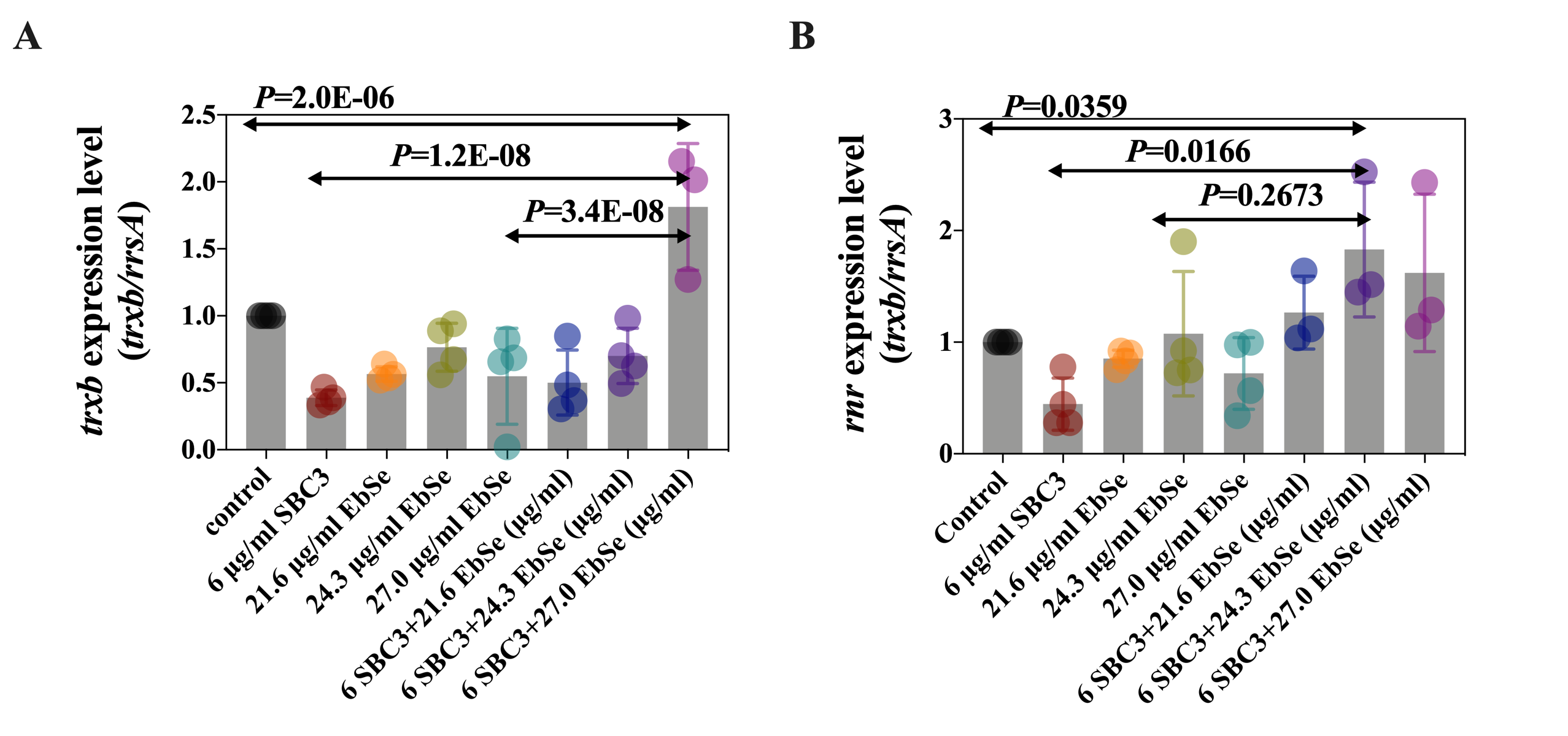

Supplement: Supplementary file 1 [file Image3.TIFF]

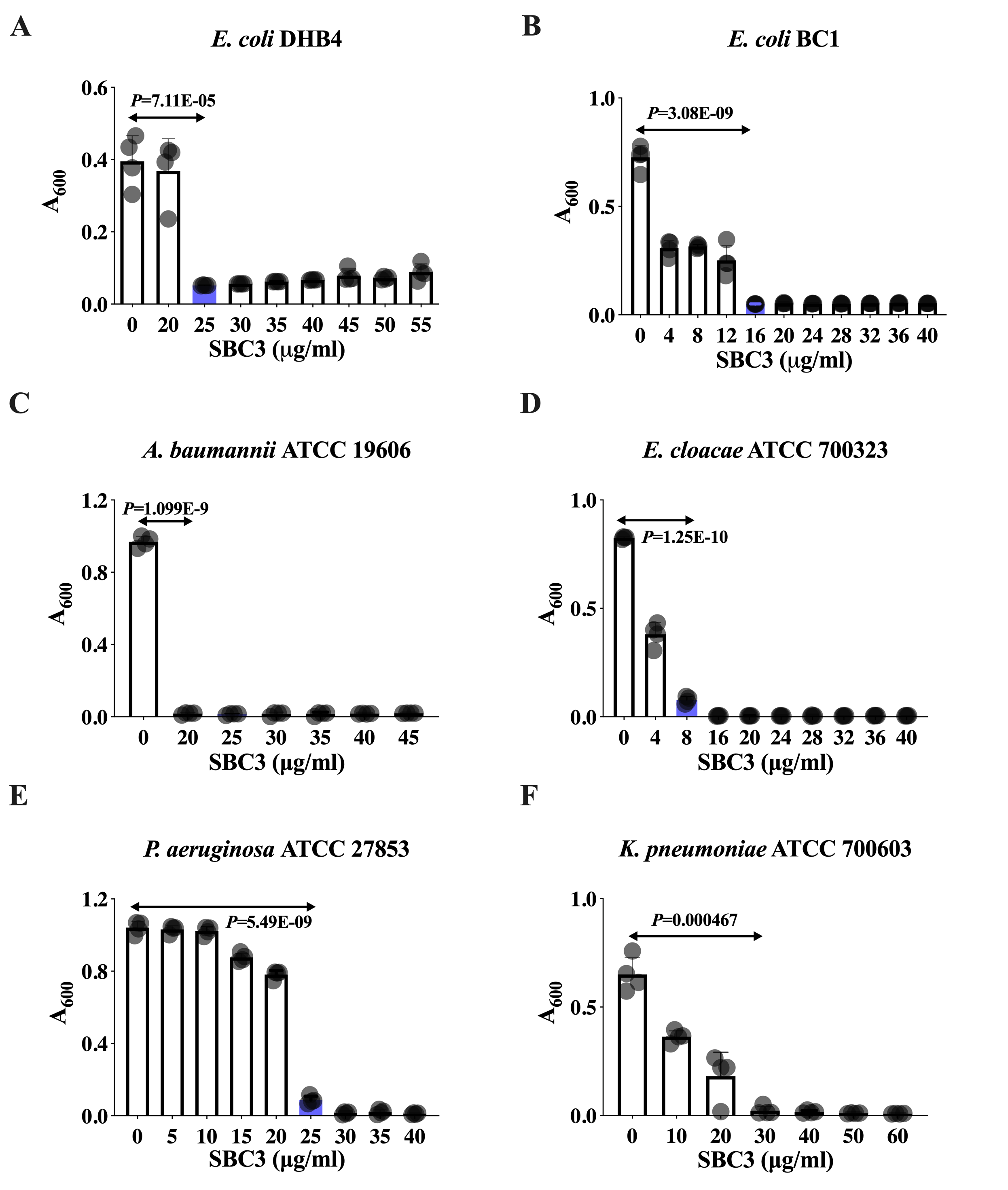

Supplement: Supplementary file 2 [file Image1.TIFF]

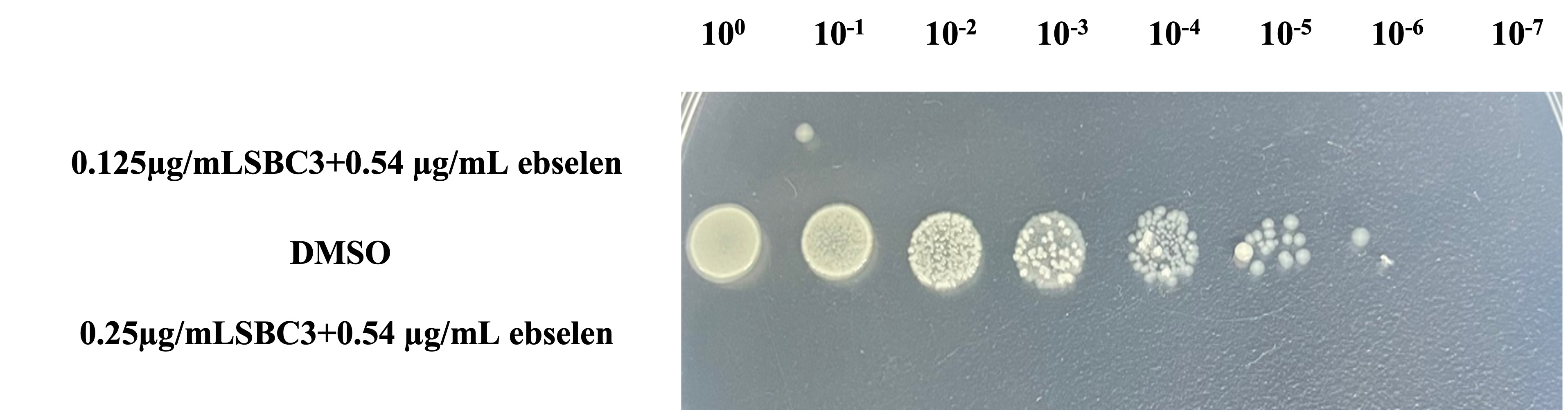

Supplement: Supplementary file 3 [file Image2.JPEG]
