# Supplementary material for: The synergistic activity of SBC3 in combination with Ebselen against Escherichia coli infection
Source: Front Pharmacol. 2022 Dec 15;13:1080281. doi: 10.3389/fphar.2022.1080281 (PMC9797518; doi:10.3389/fphar.2022.1080281)
Supplement: Supplementary file 4 [file DataSheet1.pdf]

Table S1 Primers for qPCR

Genes	Proteins	Forward (5'-3')	Reverse (5'-3')
rrsA	16S rRNA	*ctcttgccatcggatgtgccca*	*ccagtgtggctggtcatcctctca*
trxa	Trx1	*gactctgctgctgttcaa*	*aactctttcaactgacctttag*
trxb	TrxR	*acggttttgcagatccacct*	*cctggcgatccaaacgatct*
grxa	GR1	*ttagtgatcccttccgcacg*	*tttttggtcgttcgggttgc*
rnr	RNase R	*gtttatgggtcgcagttgcc*	*aacacaggccgttagagagc*

Table S2 *S* degree for the antibacterial activity of SBC3 and Ebselen

Bacteria	F_{00}	F_{0y}	F_{x0}	F_{xy}	***S* degree**
E. coli DHB4	0.50285	0.4901	0.446975	0.049725	0.767459
E. coli BC1	0.427133	0.426533	0.9332	0.0486	0.805481
A. baumannii ATCC19606	0.9329	0.9217	0.904075	0.668025	0.241394
E. cloacae ATCC700323	0.806725	0.833075	0.64785	0.047825	0.770009
K. pneumoniae ATCC700603	0.6964	0.57175	0.583275	0.050275	0.615449
P. aeruginosa ATCC27853	0.9243	0.97315	1.129625	0.04985	1.2328>1.0
